# Age-Adjusted Schedules of Venetoclax and Hypomethylating Agents to Treat Extremely Elderly Patients with Acute Myeloid Leukemia

**DOI:** 10.1155/2022/2802680

**Published:** 2022-04-26

**Authors:** Aaron M. Lee, Aaron M. Goodman, James K. Mangan

**Affiliations:** ^1^Department of Medicine, Internal Medicine Residency Program, University of California San Diego, La Jolla, CA, USA; ^2^Department of Medicine, Division of Blood and Marrow Transplantation, Moores Cancer Center, University of California San Diego, La Jolla, CA, USA; ^3^Moores Cancer Center, University of California, San Diego, 3855 Health Sciences Drive, Mail Code 0960, La Jolla, CA 92093, USA

## Abstract

Acute myeloid leukemia (AML) is associated with particularly poor outcomes in the elderly population, in whom the disease is most prevalent. BCL-2 has been identified as an antiapoptotic protein and promotes survival of leukemia stem cells. Recently, the United States FDA has approved venetoclax, a selective oral BCL-2 inhibitor, for use in conjunction with hypomethylating agents (azacitidine or decitabine) or low-dose cytarabine as a first-line treatment option for those AML patients ineligible for standard induction chemotherapy. However, there are nuances and challenges when using this regimen in the extremely elderly AML patients. Given the widespread adoption of this regimen and increasing prevalence of patients who are well into their 80 s, it is important to evaluate and understand how to safely use this regimen in this so-called “extremely elderly” population. We present here 3 case studies involving AML patients >85 years of age who were treated with venetoclax plus HMA and provide clinical knowledge on how this population should be appropriately managed.

## 1. Introduction

Acute myeloid leukemia (AML) commonly affects the elderly, with a median age at diagnosis of 67-68 years. Elderly patients (typically defined as >60 years old) often have poor responses to typical cytotoxic and intensive induction chemotherapy, given the propensity for adverse genomic features, drug-resistant phenotypes, and more comorbidities and compromised organ function than younger patients. Treating this population is a balancing act; low-intensity regimens often prove safe but ineffective, while intensive chemotherapy is associated with excess morbidity and mortality. For some time now, these elderly patients deemed unfit for induction chemotherapy have received low-intensity regimens typically consisting of monotherapy with a hypomethylating agent (HMA) or low-dose cytarabine, producing modest results and suboptimal outcomes.

In November 2018, venetoclax in combination with a hypomethylating agent (HMA) was granted accelerated approval by the FDA for AML patients ineligible for intensive chemotherapy or adults >75 years old. This has represented a major advancement in treatment of AML in the elderly. Several clinical trials studying venetoclax plus HMA showed tolerable safety profile as well as favorable response rates in patients with AML (Table 1). Dinardo et al. in a randomized phase III trial showed that in patients ineligible for standard induction therapy, use of venetoclax with decitabine or azacitidine led to overall survival advantage and higher incidence of remission compared to those who received azacitidine alone [1]. Venetoclax in conjunction with an HMA has now been widely used for those patients not fit for standard induction chemotherapy. While the median age in these trials ranged from 68 to 76 years, additional information on using these regimens to manage older, “real-world” patients, particularly those over 85 years of age, would be helpful to clinicians in clinical practice. There remains a paucity of data on how to best manage these extremely elderly AML patients with venetoclax plus HMAs.

In this manuscript, we use three case studies to showcase how we treat AML in the extremely elderly. We will discuss our clinical experience and provide expert recommendations on treatment and management.

## 2. Case Reports

### 2.1. Patient 1

Patient 1 is an 87-year-old male who presented with fatigue and intermittent shortness of breath. The WBC count was 84,900 with a large number of circulating myeloblasts. Cytogenetic and molecular details are listed in Table 1.

The patient was admitted to the hospital and started on hydroxyurea for cytoreduction. Venetoclax and azacitidine were initiated when his WBC count dropped below 35,000. Due to high risk of tumor lysis, venetoclax was dose-escalated from 100 mg on day 1 to 400 mg by day 3 and was continued on 400 mg daily through day 21. Azacitidine was given intravenously at 75 mg/m^2^ on days 1–7. His inpatient course was complicated by febrile neutropenia with a negative infectious workup and deep cytopenias requiring transfusion support.

Bone marrow biopsy on day 22 showed a hypocellular marrow with <5% blasts. His counts were allowed to recover, and he met criteria for complete remission after cycle 1. He subsequently received 10 additional cycles of therapy. He was maintained on venetoclax 400 mg daily on days 1–14 during the additional cycles with full-dose azacitidine, and cytopenias were managed with rare blood transfusions and occasional granulocyte colony stimulating factor (G-CSF). The patient's course was later complicated by Sweet's syndrome, believed to be secondary to G-CSF, at which time azacitidine and venetoclax were held. Repeat bone marrow showed his AML had relapsed, and he was restarted on azacitidine alone. He had a stable disease on azacitidine for 6 months before progressing with leukocytosis and circulating blasts. Next-generation sequencing revealed that the patient had acquired a FLT3 ITD mutation that was not previously present. He was switched to monotherapy with the FLT3 inhibitor gilteritinib and had a hematologic response before passing away from complications of AML 25 months after his original diagnosis.

### 2.2. Patient 2

Patient 2 is a 92-year-old male who presented with recurrent pneumonia and a biopsy-proven right lower lobe lung adenocarcinoma and was incidentally noted to be pancytopenic. A bone marrow biopsy was markedly hypocellular, with 43% myeloid blasts in scattered clusters. Cytogenetic and molecular data are displayed in Table 2.

The patient was referred to Thoracic Oncology and underwent successful radiation in 10 fractions of a stage I lung adenocarcinoma, and given his good performance status otherwise, he was admitted to the hospital and started on treatment with intravenous azacitidine (75 mg/m^2^ on days 1–7) and venetoclax 400 mg daily on days 1–21. The patient tolerated induction well with minimal adverse effects, without neutropenic fever, and only mild weight loss and deconditioning. Repeat bone marrow biopsy on day 21 showed a markedly hypocellular marrow with a small myeloid blast population (3%), and he ultimately had good count recovery.

Following induction, the patient continued to tolerate the treatment well, with only symptoms of fatigue and occasional nausea. He did have persistent cytopenias, necessitating occasional transfusions and routine twice weekly G-CSF injections. The patient underwent 9 more cycles of therapy, including 5 cycles with azacitidine alone and 4 cycles with venetoclax given for only 14 days at the full dose of 400 mg daily. After 10 cycles of therapy, the patient showed evidence of relapsed disease and progression and passed away 17 months after the initial diagnosis.

### 2.3. Patient 3

Patient 3 is an 85-year-old male who had a transformation to acute myeloid leukemia after a yearlong history of MDS with multilineage dysplasia. He presented with leukocytosis and circulating blasts, and his bone marrow biopsy confirmed transformation to acute leukemia with both myeloid and T-lymphoid marker expressions. There was extensive marrow involvement. Cytogenetic and molecular details are displayed in Table 1.

The decision was made to start decitabine with venetoclax, and the patient was admitted for induction therapy. During induction, the patient received dose-reduced venetoclax 100 mg daily for 21 days due to concern for drug-drug interactions, given the concomitant use of posaconazole for fungal prophylaxis. A bone marrow biopsy following cycle 1 showed a cellular marrow with reduction of blasts from 80% down to 14%, with a normal absolute neutrophil count and stable thrombocytopenia (platelet counts stable around 20,000, compared to 25,000 on admission), and he was discharged without further complications.

On follow-up, the patient was continued on decitabine and venetoclax, receiving only 14 days of dose-reduced 100 mg venetoclax for each cycle, given persistent cytopenias. His course was complicated by anemia and fatigue, and the patient received G-CSF during periods of neutropenia between cycles and transfusions as needed. For cycles 2 and 3, the patient received a shorter dose of 14 days of venetoclax due to anemia. Bone marrow biopsy after cycle 3 showed ongoing reduction in the blast count (9% blasts). Cycle 4 was with decitabine alone, and the patient developed leukocytosis and a bacteremia, so after stabilization, cycle 5 was resumed with decitabine and venetoclax once again. Following cycle 5, the patient developed GI bleeding in the setting of thrombocytopenia. The patient was managed supportively on hydroxyurea and transfusion support, ultimately transitioning to hospice care, 6 months after his original diagnosis with AML.

## 3. Discussion

We present three cases of extremely elderly patients (>85 years old) with AML who have been treated with venetoclax and a hypomethylating agent (azacitidine or decitabine). There are several tools that have been used to assess fitness of elderly patients for AML therapy, some of which have been described by Min et al. [7]. We did not use a formal tool to select patients, but all patients selected were ambulatory, did not have any cognitive impairment, and had excellent family support and transportation to and from our center. The ECOG performance status was at least 2 at the time of treatment initiation. After receiving therapy, all three patients had bone marrow responses and two of the three patients completed over 10 cycles of therapy. All patients were treated with the full therapeutic dose of venetoclax when given in combination with HMA. After achieving initial response, we did modify subsequent cycles of VEN/HMA to use VEN only for 14 days instead of 21 and occasionally omitted venetoclax cycles and treated with HMA alone, all in an effort to manage cytopenias and avoid significant complications such as neutropenic fevers while the patients were on active treatment. Fungal prophylaxis and bacterial prophylaxis were used while the patients were neutropenic. Importantly, the patients were motivated for therapy, and two of three maintained a good quality of life while being treated. Patient 1 lived for 25 months after the initial diagnosis, and patient 2 survived for 17 months after the initial diagnosis. This case series illustrates that treatment with HMAs and venetoclax is feasible in the extremely elderly population and age alone should not rule out such patients from receiving active antileukemia therapy.

## Figures and Tables

**Table 1:** Clinical trial outcomes in AML patients using venetoclax plus hypomethylating agents.

Study	Phase of study	Patient population	Number of patients	Treatment and dosing used	Median age, years (range)	# patients >75 years old	Response rate (CR + CRi)	CR	Median event-free survival	Median duration of CR + CRi	Median overall survival	Grade 3/4 adverse events
Dinardo et al., *Lancet Oncol*, 2018 [2]	1b	Age >65, treatment naïve, ineligible for standard induction	57	3 + 3 dose escalation; venetoclax-group A: target doses 400, 800, and 1200 mg/m^2^; group B: target doses 400, 800, and 1200 mg/m^2^; group C: target dose 400 mg/m^2^; azacitidine-75 mg/m^2^; decitabine-20 mg/m^2^	Group A: 74 (71.5–79.0); group B: 75 (71.0–80.0); group C: 74 (69.0–79.5)	39	61.4% (35/57)	24.6% (14/57)	Not reported	Group A: 8.4 mos; group B: 12.3 mos; group C: 4.3 mos	12.3 months (all groups)	Thrombocytopenia; febrile neutropenia; neutropenia
Dinardo et al., *Blood*, 2019 [3]	1b	Age >65, treatment naïve, ineligible for standard induction	145	Venetoclax-ramp-up C1 from 20 (escalation phase) or 100 (expansion); target doses: 400, 800, and 1200 mg/m^2^; in escalation expansion phase: 400 mg and 800 mg; azacitidine-42 mg/m^2^, d1-7; decitabine-20 mg/m^2^, d1-5	74 (65–86)	52	67%	37%	Not reported	11.3 months	17.5 months	Febrile neutropenia; anemia; thrombocytopenia; neutropenia; pneumonia
Dinardo et al., *NEJM*, 2020 [1]	3	Ineligible for intensive induction, age >75, all treatment naive	286 in variable group	Aza 75 mg/m^2^, d1-7; VEN 400 mg daily, d1-28 w/3 day ramp-up C1	76 (49–91)	Not reported	66.4%	36.7%	9.8 months	17.5 months	14.7 months	Thrombocytopenia neutropenia; febrile neutropenia; anemia; leukopenia
Maiti et al., *Blood*, 2018 [4]	II	Age >69, ineligible for OR relapse/refractory AML	48	VEN days 1–28, cycle 1, D1-21; C2 and onwards VEN 200 mg PO daily in pts needing cyp3a4 decitabine-20 mg/m^2^ IV daily D1–D10 until CR, followed by 5-day cycles	71 (22–82)	Not reported	71% (34/48)	CR alone not reported	Not reached	Not reached	Not reached	Infections; neutropenia; tumor lysis syndrome
Wei et al., *Journal of Clinical Oncology*, 2019 [5]	Ib/II	Age >60, treatment naïve, ineligible for intensive chemo	82	Venetoclax-initial 50 or 100 mg, titrated up to the target dose; phase II recommended dose: 600 mg; 800 mg in phase Ib w/prolonged myelosuppression; low-dose cytarabine-20 mg/m^2^ daily SC injection D1–10, 28-day cycles	74 (63–90)	40	54%	26%	Not reported	8.1 months	10.1 months	Cytopenia, febrile neutropenia; infections
Wei et al., *Blood*, 2020 [6]	3	Adults ≥18 y/o w/ND AML ineligible for intensive chemo (38% had secondary AML, and 20% had received prior hypomethylating agent treatment)	210	Venetoclax + LDAC	76 (33–93)	82	48%	27%	4.7 months	Not reported	7.2 months	Febrile neutropenia; neutropenia; thrombocytopenia

**Table 2:** Clinical characteristics of three extremely elderly AML patients treated with venetoclax and azacitidine.

	Age at diagnosis	Medical comorbidities	Blood count at bone marrow diagnosis	Cytogenetics	Next-generation sequencing	Blast (%) in the marrow at diagnosis	Bridging therapy used prior to venetoclax plus HMA	Hospitalized for induction?	Complications from induction	Response to induction	Fungal prophylaxis during induction	Progression-free survival	Postinduction complications
1	87	Melanoma (*in situ*, resected); hyperlipidemia; hypertension	WBC: 84,900; Hgb: 9.3; platelet: 81; ANC: 5943	Karyotype: normal; FISH: normal	SF3B1 K666 N (VAF 47%), FLT3 D835Y (VAF 45%), and RUNX1 D93Afs ∗ 31 (VAF 42%)	95	Hydroxyurea	Yes	Neutropenic fever; atrial fibrillation with rapid ventricular response; upper extremity phlebitis	CR	Micafungin	13 months, relapse following cycle 11	Sweet's syndrome following C11
2	92	Lung adenocarcinoma; prostate cancer (*localized*); hypertension	WBC: 0.4; Hgb: 8.6; platelet: 72; ANC: 136	Abnormal karyotype; deletion 6q; FISH: loss of 6q (33%) and 7q (29%)	Mutations in DNMT3A, SRSF2, RUNX1, BCOR, TET2, and BCR-ABL neg	43	None	Yes	None	CR	Micafungin	13 months	Neutropenic fever following C10; COVID-19 pneumonia
3	85	Prostate cancer (*localized*); minimal change disease; hypertension; hyperlipidemia	WBC: 38.3; Hgb: 7.6; platelet: 25; ANC: 5745	Karyotype: abnormal; deletion 20q; gain of *X* chromosome in two clones; FISH: diminished D20S108 (20q12) hybridization in 65% of cells	Mutations in RUNX1, SMC3, and TET2	80	Hydroxyurea	Yes	Tumor lysis syndrome	Partial response	Posaconazole	4 months on therapy, progression during cycle 4	GI bleeding and bacteremia following C5

Patient 1: C1 venetoclax 400mg 3 day ramp up to D21 + azacitidine 75 mg/m^2^ D1-7. C2 venetoclax 400mg D1-21, azacitidine 75 mg/m2 D1-7. C3 venetoclax 400mg D1-14, azacitidine 75 mg/m^2^ D1-7. C4-C5 azacitidine 75 mg/m^2^ D1-7, venetoclax held due to cytopenia. C6-C11 Ven 400mg D1-14, azacitidine 75 mg/m2 D1-7. C12-17 azacitidine only. Gilteritinib thereafterPatient 2: C1 venetoclax 400mg (no escalation) D1-21, azacitidine 75 mg/m^2^ d1-7. C2-3 venetoclax 400 D1-14 (avoid cytopenia), azacitidine 75 mg/m^2^ D1-7. C4-5 azacitidine only secondary to cytopenias. C6-7 venetoclax 1-14d, with azacitidine. C8-10 azacitidine only. Patient 3: C1-3 venetoclax 100mg daily 1-21, Decitabine 20 mg/m^2^ d1-5. C4 Decitabine 20 mg/m^2^ d1-5. C5 venetoclax 100 mg daily, Decitabine 20 mg/m^2^ d1-5.

## Data Availability

The data that support the findings of this study are available from the corresponding author upon reasonable request.
